# The Impact of Online Media on Parents’ Attitudes toward Vaccination of Children—Social Marketing and Public Health

**DOI:** 10.3390/ijerph17165816

**Published:** 2020-08-11

**Authors:** Boban Melovic, Andjela Jaksic Stojanovic, Tamara Backovic Vulic, Branislav Dudic, Eleonora Benova

**Affiliations:** 1 Faculty of Economics, University of Montenegro, 81000 Podgorica, Montenegro; bobanm@ucg.ac.me (B.M.); tassabacc@ucg.ac.me (T.B.V.); 2 Faculty of Culture and Tourism, University of Donja Gorica, 81000 Podgorica, Montenegro; andjela.jaksic@unimediteran.net; 3 Faculty of Management, Comenius University in Bratislava, 82005 Bratislava, Slovakia; eleonora.benova@fm.uniba.sk; 4 Faculty of Economics and Engineering Management, University Business Academy, 21000 Novi Sad, Serbia

**Keywords:** online media, social media, vaccination, social marketing, public health

## Abstract

The aim of this paper was to investigate the level of influence of online media on the parents’ attitudes toward vaccination of children in three countries of the Western Balkans—Montenegro, Serbia, and Bosnia and Herzegovina, in order to use the potentials of this form of communication effectively and efficiently. Online media are a critical factor of influence on the formation of attitudes in many areas of modern society, which is why their proper use plays an important role in strengthening vaccine confidence and which may further contribute to improvement of public health. On the other side, having in mind the fact that communication is an integral part of marketing, it is clear that social marketing has an extremely important role regarding the analyzed topic, especially because of the fact that social marketing activities tend to change or maintain people’s behavior for the benefit of individuals and society as a whole. For the purpose of this research, a conceptual model was developed. Quantitative research was conducted online in the first quarter of 2020 using the survey method. Statistical analysis was applied to data collected from 1593 parents in the analyzed countries. The relevance of the hypotheses was tested using standard statistical tests, ANOVA test, eta coefficient, and logistic regression. The research showed that all analyzed variables from the model have a significant impact on the parents’ attitudes toward the vaccination of children and that they correlate with the degree of trust in vaccines. The results also approved that online media have a significant influence on the formation of parents’ attitudes toward the vaccination of children (obtained values of eta coefficient η^2^ = 0.216, η^2^ = 0.18, η^2^ = 0.167, η^2^ = 0.090, reliability Cronbach’s Alpha 0.892), which confirms the importance of the use of social marketing in order to direct communication properly and to strengthen the level of trust in vaccines. Additionally, the results of logistic regression showed that the following groups of parents are particularly vulnerable to the influence of online media on attitudes toward vaccines: women, parents of younger age (“millennials”), and parents who are in common law marriage, as well as parents who have more children. In addition, the results showed that there is no statistically significant difference in the attitudes of parents in the observed countries (η^2^ = 0.000, F = 0.85). Based on the results of the research, the authors suggest that decision makers should pay more attention to modern forms of online communication and social marketing in order to use their potential for improvement of public health, as well as avoid the harmful impact that certain forms of communication may have on the formation of attitudes and loss of confidence in vaccines. The findings provide an important contribution for public health policy makers to identify and understand properly the impact of online media and social marketing and thus to better adapt their initiatives to changes in modern society.

## 1. Introduction

Today, people may find a lot of different information regarding public health online. Research studies have shown that online sources represent a well-established and important site of health-related information seeking behavior [1,2], and, moreover, have a significant role in shaping health behaviors [3]. For example, one in three adults in the United States tries to diagnose a medical condition online [4]. Online sources have thus become the primary source of information in the 21st century, which is especially present in the field of medicine and public health. Through online information, almost everyone has access to numerous information with just few clicks. In other words, online media represent a critical factor of influence toward attitudes in different areas, which is why the proper use of this form of communication also plays an important role in improvement of public health. However, online sources also contain misinformation that may negatively affect attitudes and behavior and, as such, may have extremely harmful effects on public health [5]. In addition, health misinformation, which is against established medical understanding [6], may be widely distributed in order to reach a large population in a short time in the digital age [7]. This is extremely important because previous studies have shown that many parents mostly receive vaccination information through online sources [8].

Although vaccination is considered to be the most effective and cost-effective way of preventing the contraction of an infectious disease [9,10], there are numerous controversies about vaccines. First of all, it is important to point out that vaccination is recognized as an integral part of public health policies and each country implements vaccination requirements in order to achieve satisfactory vaccination coverage. Avoidance of vaccination by parents directly leads to a lower vaccination rate among their children, increasing the social risk of infection [11]. In line with the above, a large number of previous studies have focused on the benefits of vaccinating children [12,13], which further positively reflects on strengthening trust in vaccines. Thus, researchers in many countries have emphasized the cost-effectiveness of vaccination in preventing disease [14,15,16] and this issue is especially important in less developed countries. Despite the trend of increasing vaccination rates around the world, many factors may influence the formation of negative attitudes, especially in developing countries, such as the countries of the Western Balkans region. Namely, these countries, most often, have a lower level of development of the health system and public health policy, which further reflects the influence of online media on the formation of attitudes and level of trust in vaccines, which is one of the motives of this research. This problem is faced not only by developing countries but also by developed countries. The issues related to vaccines are increasingly politicized today. An international study on attitudes towards vaccination has shown that, although overall confidence in vaccines is positive, it is the lowest in the European region [17].

Although there is a certain number of studies on trust in vaccines [18,19,20], it is often pointed out that there is a lack of such research, especially in less developed countries, which is why this issue continues to cause a lot of controversy. Namely, despite the scientific consensus that vaccines are safe and effective, there are still unconfirmed claims that doubt their safety [21]. In line with the above, some research studies show that public confidence in vaccines is increasingly lost, and there are more and more people who are beginning to question the safety of vaccines, changing the recommended vaccination schemes, or even rejecting vaccination [22,23,24]. This problem is especially pointed out when it comes to less developed countries, such as most of the countries of the Western Balkans. In recent years, vaccines have been “notorious” in these countries. This especially refers to the measles-mumps-rubella (MMR) vaccine because of its potential association with autism [25]. This creates a dilemma among parents whether to vaccinate their children or not, not only when it comes to this but also to other vaccines. The confirmation for this statement is the fact that there is a large number of cases that the pediatrician informed the health inspector that the parents refused to vaccinate or revaccinate the child. In order to increase the number of vaccinated children, the countries of the Western Balkans often prescribe penalties. Thus, for example, parents in Montenegro, although in a dilemma, are obliged to vaccinate their children against ten infectious diseases, and, if they do not do so, they must pay a fine which is prescribed by the Law on Protection of the Population from Infectious Diseases [26]. However, there is another indirect sanction for parents who do not vaccinate their children. Namely, an unvaccinated child cannot be enrolled in a kinder garden or school, and the parents should provide medical certificates in order to confirm that the child is vaccinated. However, according to the latest data of the Institute of Public Health from the February 2020, 8000 children in Montenegro of ages from three to five did not receive mandatory vaccines [27]. In the past two years, the Directorate for Inspection Affairs has filed over 177 misdemeanor charges against parents who did not vaccinate their children according to the compulsory immunization calendar, while courts imposed 150 fines worth of 15,000 euros in total while the other 27 parents received a reprimand [28]. The situation is similar in other countries of the Western Balkans, especially in Serbia and Bosnia and Herzegovina, which are included in this research. All this leads to the conclusion that a large number of parents have a dilemma about vaccination and that they have distrust in vaccines, which is why they often do the research about vaccines for themselves in order to make a decision. Distrust in vaccines served as one of the motives for this study.

So, in many countries, health experts state that there is a trend of mistrust when it comes to vaccines, and thus a refusal to use them. The World Health Organization (WHO) has included this trend in one of the 10 threats to world health in 2019 [29]. At the same time, it should have in mind that a number of studies highlight the negative aspects of vaccination, which are very often the result of media influence. A health scare, or panic created by the media in relation to health issues, has been shown to increase people’s need for information and for people to begin to question traditional sources of information as trustworthy [30]. Furthermore, research shows that vaccination rates vary depending on the use of the mass media [31], especially online media, which is the dominant form of communication in most countries. In line with the above, some studies have shown that more and more parents are searching for vaccination information on various online sources [8].

As previously pointed out, online media are a critical factor of influence on the formation of attitudes in many areas of modern society, which is why their proper use plays an important role in increasing trust in vaccines, and thus improving public health. Online media include various forms, such as medical websites, social networks, portals, blogs, forums, etc., and research shows that some of them like social media have the capacity to influence and shape public opinion regarding vaccination in a viral manner—both positively and negatively [32]. In this context, it is extremely important to analyze online media as a part of marketing communication and social marketing, which also has an important role in the improvement of public health. Namely, social marketing is an approach used to develop activities aimed at changing or maintaining people’s behavior for the benefit of individuals and society as a whole. Social marketing, through its various forms and strategies, plays a significant role in the field of medicine and public health [33]. In this way, social marketing influences parents’ attitudes and better understanding of online media and marketing communications and decision makers as an important factor in strengthening trust in vaccines and improving public health. More specifically, it is very important that both parents and decision-makers understand social marketing, as well as to understand how particular forms of marketing communication, such as online media, influence perceptions, and attitudes about vaccination of children [31], which is one of the motives of this research. This is because, as mentioned above, despite the existence of numerous studies that explain the benefits of vaccination, there are still many conflicting views on pro-vaccine and anti-vaccine [34], especially having in mind more intensive use of online media and marketing campaigns in modern age. Research studies show that anti-vaccine articles are more likely to be shared, commented on, and reacted to online than pro-vaccine messages [35]. Online anti-vaccine messages may lead parents to question the safety of vaccine, distrust health professionals, and seek non-medical vaccine exemptions [36,37]. Regarding this matter, several studies have been conducted in order to analyze how online information influence parents’ attitudes and decisions about vaccination. Some studies have focused on specific vaccines, while others have been general [8]. According to one of the studies, “the most recent statistics available show 16% of seekers searched online for vaccination information and 70% say what they found affected by their treatment decisions” [38].

In line with the above, research studies around the world show that media exposure may significantly facilitate a change in parents’ behavior [21,39], which is especially important when it comes to using online media. This would contribute to the strengthening of trust in vaccines, as well as to the improvement of public health, through adequate online communication and various forms of social marketing. It should have in mind that, in addition to the positive effects, online media may also use groups to get people to oppose vaccination, raising skepticism about the scientific evidences regarding the risks and benefits of vaccines [40]. Online media, especially web pages against vaccination, are widely spread on Internet [41] and, in many countries, may be more compelling sources of information than vaccination sources [42]. People have been shown to be more responsive to personal stories than statistics [43], which means that online vaccine sources and their personal stories may create a stronger emotional response for readers than official health online sites with statistics and arguments.

Thus, media in general and specially the online media, have significantly contributed to widespread public distrust of vaccines in many countries around the world [44], and countries in our region are no exception. In fact, the dissemination of negative information about immunizations has been increased by the progress of certain forms of online resources, such as individual social networks (Facebook and Twitter) [44,45]. Since 2013, the World Economic Forum has cited mass digital misinformation among the major threats to our society [46]. Recent studies emphasize that the spread of misinformation is the result of a paradigm shift in content consumption caused by the advent of social media. In fact, the platforms of particular forms of online media, such as Facebook or Twitter, have created a direct path for users to produce and consume content, changing the way people inform themselves [32,47,48] and form attitudes. It is often discussed that online media, and especially social media, have important role in creating hesitancy [10] and fear in parents and encouraging them to avoid vaccination. Many of these fears come from information that parents find online and many of these sources not only propagate unproven claims regarding vaccines but may also undermine the physician-family relationship by challenging parents’ trust in the medical professionals [49].

On the other hand, many parents from many countries who have decided not to vaccinate their children have done their own (online) research. Research studies show that parents seeking for information about vaccine risk will find more online sources that are against the vaccine, compared to parents seeking information about the benefits of vaccines [50]. This means that it is likely that parents who are worried about vaccination will find online sources to confirm their fears. So, today the information is widely spread through different forms of online marketing especially social media and networks. For example, a quick Facebook search provided more anti-vaccination groups from around the world. Anti-vaccine content exists in many of the vaccine-related top Google search results [42,51]. By doing a Google search on the key term “vaccine refusal”, 3,340,000 results could be found [8]. It may be concluded that despite all the advantages of vaccination, there is still a strong resistance in form of anti-vaccine movements, which are on the one side the result of mistrust, and on the other of the strong influence of the media. It is important to emphasize that vaccine-related misinformation, which is often spread via the Internet by vaccine groups [51], may be the most commonly distributed health-related misinformation [52].

Relying on the results of previous research and the observed literary gap, the authors wanted to conduct a study that would target the impact of online media on parents’ attitudes towards vaccination of children, as well as the impact of other characteristics (gender, age, country of origin, etc.), in order to direct social marketing activities to strengthening trust in vaccines, thus improving public health. So, in order to discover, identify and understand the relationship between online media and parents’ attitudes toward children’s vaccination, especially from the country of respondents’ origin (Montenegro, Serbia, and Bosnia and Herzegovina), this paper tends to fill the gap compared to previous studies. Therefore, the aim of the paper was to investigate the level of influence that online media, as a form of marketing communication, have on the formation of parents’ attitudes toward the vaccination of children, that is trust in vaccines in analyzed countries of the Western Balkans region, as well as a role of social marketing in strengthening trust in vaccines and improvement of public health.

The paper is organized into five sections. Following the abstract, in the first section, a review of the results of previous research regarding the vaccination and online media was made, as well as the literature overview in which the motive for this research was found. This section contains an analysis of key aspects of vaccination, arguments pro and against vaccines, vaccines trust, the role of online media, and the importance of social media in strengthening trust in vaccines, as well as the influence of the country development on these questions. This segment also refers to materials and methods and includes a description of research methodology, i.e., data collection and simple, measures and instrument validation. The next part presents the results of research, while the fourth part represents the discussion of the results. Finally, the paper concludes with concluding remarks, a review of the implications, and recommendations for future research studies.

## 2. Hypotheses Development, Materials, and Methods

Based on the relevant literature and using data obtained from empirical research in three countries: Montenegro, Serbia, and Bosnia and Herzegovina, several hypotheses were developed in order to investigate the relationship between the analyzed variables and parents’ attitudes toward vaccination of children, especially from the aspect of online media.

As previously noted, existing research supports the thesis that some of the demographic characteristics may be important in forming parents’ attitudes toward vaccination of children. Thus, for example, Brown et al. [53] emphasize the gender and age of parents, while Anderberg et al. [54] point out that decisions about vaccinating children significantly depend on the level of education of parents, because a higher level of education is translated into a higher awareness or the information being perceived differently. Similarly, Walsh et al. [55] relate the age and education of parents. Furthermore, the aim of some research in this area is to determine the extent to which parents’ attitudes towards immunization affect coverage (number of vaccinated children) and to assess the level of parents’ knowledge about immunization [56]. In addition, a number of these studies highlight the country’s level of development as an impact factor, and these studies do not analyze this. In addition, the adequate application of social marketing may influence the change of behavior [57], i.e., it may influence the strengthening of trust, considering the previously mentioned characteristics when creating marketing communication strategies. Thus, the results of previous studies point out that gender, age of parents and their education, as well as knowledge about immunization, may be mentioned as important influencing factors, which the authors wanted to investigate in this research. In accordance with the above, the following hypotheses have been defined:

**Hypothesis** **1** **(H1).** *Identified characteristics of respondents have a significant effect on attitudes toward children’s vaccination, which reflects on the level of trust in vaccines.*

On the other hand, previous literature suggests that the online media today represent the crucial factor of influence on formation of attitudes in different areas, and that is why their proper use has important role in strengthening trust in vaccines and improvement of public health. Different forms of online marketing may have positive and negative influence [32]. Under the influence of online media, conflicting views on pro-vaccine and anti-vaccine may often be heard [34]. Thus, anti-vaccine articles are more likely to be shared, commented on, and reacted to online than pro-vaccine messages [11]. It is indisputable that media exposure influences the change of parental behavior [21,39]. Hence, the concept of social marketing has more importance and, through various forms and strategies, it plays a significant role in the field of medicine and public health [33]. Thus, in the context of the research topic, online media may be considered as a segment of social marketing, which plays an important role in improving public health. Namely, online media and other forms of marketing communication may have an impact on perceptions and attitudes toward vaccination of children [31], which encourages the importance of social marketing in order to improve public health. In accordance with the above, the following hypotheses have been defined:

**Hypothesis** **2** **(H2).** *Online media have a significant impact on parents’ attitudes toward children’s vaccination, which encourages the importance of adequate implementation of social marketing in the function of improving public health.*

In addition to demographic characteristics and the influence of online media, the authors wanted to analyze other factors that may be relevant, and which, in interaction with other factors, may have a strong influence on the formation of parents’ attitudes toward vaccination. According to this, the authors noted that the level of country’s development and policy, i.e., the measures that countries take in terms of vaccination may have a significant impact on the formation of attitudes. In this sense, a significant number of countries are trying to develop motivational measures, and we often talk about the obligation to vaccinate and penal policy in case of refusal [58]. For example, despite the implementation of vaccination regulations in Poland, as in other European Union countries, the final decision on vaccination of children is made by their parents or legal guardians [59]. On the other side, experience in the countries of our region shows that developed countries, such as Croatia and Slovenia, have similar policies, while, in countries with lower levels of development, such as Montenegro, Serbia, Bosnia and Herzegovina, there is a stricter vaccination policy, which includes high penalties and provisions in case of refusal of vaccination. In that sense, understanding the attitudes and opinions of parents toward vaccination is essential for planning and undertaking extensive and properly directed educational actions in order to prevent their indecision. Taking these measures, supported by an adequate social marketing strategy, may lead to a strengthening of trust in vaccines, as well as an improvement of public health. Starting from the fact that the countries in which the research was conducted belong to less developed countries, which have relatively similar legislation, people’s habits, and that all three countries tend to harmonize public health policies in accordance with European standards, the third hypothesis was formulated:

**Hypothesis** **3** **(H3).** *There is no significant difference in parents’ attitudes toward children’s in the analyzed countries regarding to the impact of online media.*

**Figure 1:**
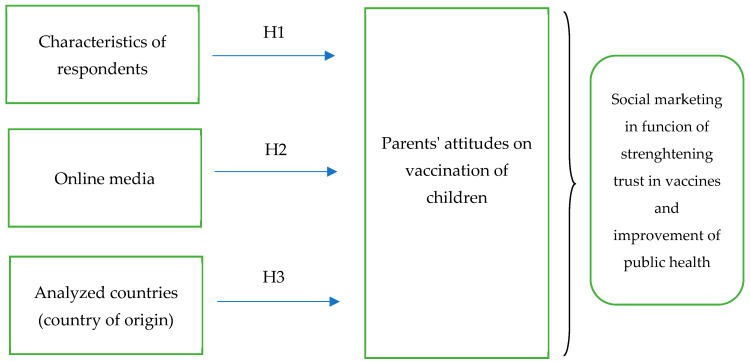
Conceptual model of research. **Source:** Authors.

The conceptual model, based on the defined hypotheses in given in the figure below (Figure 1).

Having in mind motives and goals of the research, the defined hypotheses, results of previously published research, as well as evaluations of theoretical models, the authors developed a form of a questionnaire. The questionnaire was prepared and distributed to 3031 parents in three countries (Montenegro, Serbia, and Bosnia and Herzegovina). Namely, in cooperation with preschool institutions (kindergartens) and parents’ associations, the questionnaire was transmitted online (via mailing lists and viber groups) in order to ensure the highest possible representativeness of the sample. The poll lasted for 30 days, and 1593 fully filled in polls were returned, giving the answer rate of 52.55%. This can be considered a high response rate, which is explained by the actuality of the topic itself and the parents’ interest to participate in the research. The survey was undertaken in the first quarter of 2020. The questionnaire identified 20 questions, and, for the purpose of analyze of results of the survey, according to identified criteria, 3 variables were defined. The pilot survey, which tended to examine the validity of the content of the questionnaire, was conducted in Montenegro by 15 parents. Based on their suggestions, the final form of the questionnaire was created.

Cronbach’s alpha was used to test the reliability of the study. The calculated values of Cronbach’s Alpha are at a satisfactory level and are 0.892 (the Cronbach’s alpha values adhered to the suggested minimum value of 0.6), which means that the data are suitable for further analysis [60,61]. We processed the collected data in the SPSS program (Statistics 20) and, during the analysis, we used descriptive statistics, the ANOVA test, the eta coefficient and logistic regression. Analysis of variance (ANOVA) is an analytical model for testing the significance of differences [62,63]. The advantage of this method is that the model considers all the variables, as well as their interaction. Analysis of variance is essentially a special mathematical and statistical procedure that allows testing the significance of the difference between arithmetic means from three or more samples, and within that testing the influence of one or more factors on the variability of a tested numerical feature. Furthermore, in order to further examine the importance of a certain way of using online media to form attitudes, i.e., parents’ trust in vaccines, eta-coefficient was used. The Likert scale was treated as an interval scale by placing neither in the place of neutral or moderately [64]. It is obvious that the data itself divide into two categories were the parametric tests is applicable. If the Likert scale data are treated as interval scale data, then the ANOVA test can be used. If the Likert scale data are from 1–5 with equal intervals then the midpoint already exists at 3 [65]. Therefore, the Likert scale is the same as the interval scale, with the difference in the labeling. A Likert scale, finally, label does not create any difference in the data distance since the codes are the same so the usage of parametric tests will get the best results [66].

On the other hand, logistic regression was used in order to obtain a more precise answer to the question of the relationship between the demographic characteristics of the respondents and their attitude towards vaccines based on information from online media. Logistic regression is the most commonly used in order to rank the relative importance of independent variables and to quantify the effect of their interaction [67]. The results of the research are given below.

## 3. Results

In order to determine the influence of the analyzed factors on parents’ attitudes toward children’s vaccination and their confidence in vaccines, an analysis of the characteristics of the respondents was performed using the descriptive statistics method, which is presented in Table 1.

Based on the descriptive statistics provided in Table 1, it may be concluded that the respondents are predominantly female (as much as 70.1% of the total number of respondents, which may indicate a greater interest of mothers in the mentioned topic), with the largest number between 35 and 40 years (40.7% of respondents). Respondents are married in 89.7% and possess a faculty diploma in 40.1%. The largest number of respondents is from Montenegro (44.3% of respondents, which may be justified by the fact that the research was initiated in this country) and have two children (45.1%). In terms of geographical spread, the demographic of the respondents is as follows: 705 (44.30%) respondents are from Montenegro and 520 (32.60%) from Serbia, while 368 (23.10%) are from Bosnia and Herzegovina.

Furthermore, these characteristics were correlated with the degree of trust that parents have in vaccines, which is shown in Table 2.

The greatest dispersion of data about the average value of the attitude that they do not trust in vaccines was noticed in the question related to the age of the respondents. The standard deviation of this characteristic is 1359. Thus, the age of a randomly selected sample deviates from the average value of all respondents in the amount of 1359 points on the Likert scale, provided that these subjects do not trust vaccines. High data dispersion was also noted for the level of education of respondents in this category of trust in vaccines because the value of the standard deviation is 1006. The two highest values in category I, totally believe, are for education (1473) and age (1191). The analysis of other categories of trust in vaccines also showed that the results are the most dispersed for the level of education and for age because standard deviations have the highest values for these two characteristics of the respondents. The most homogeneous answers refer to the gender and marital status of the respondents.

**Figure 2:**
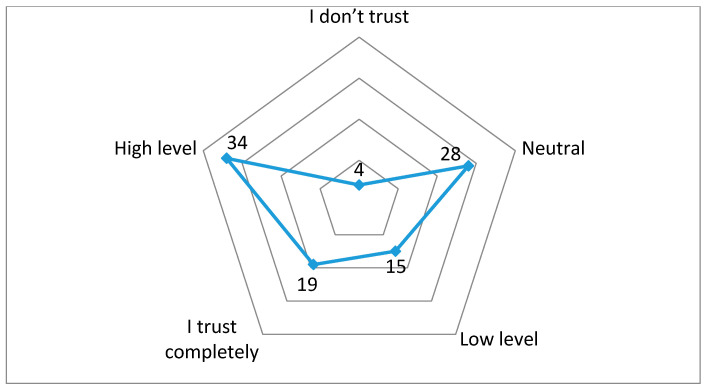
Trust in vaccines.

Based on the contingency table, a graphical presentation (Figure 2) of the participation of individual categories of respondents in terms of characteristics, such as gender, country of origin, age, marital status, and number of children, in combination with their attitude about trust in vaccines was created.

The analysis of the contingency table showed that the largest number of respondents have high confidence in vaccines (34%), when their characteristics, such as gender, country of origin, age, marital status, and number of children, would be analyzed. However, about one-fifth of respondents do not trust or have a low level of trust in vaccines, which means that certain actions must be taken in order to improve this situation. This was the motive to focus on discovering the reasons why parents do not trust vaccines in one part of the research in order to create set of recommendations which would contribute to the strengthening of trust in vaccines. 

Furthermore, characteristics of respondents have been correlated with the attitudes of parents towards vaccines, as shown in Table 3. Since the survey was conducted in three countries, three groups of respondents were available for testing, so it was possible to apply the ANOVA test.

The starting hypothesis of the ANOVA test indicates the equality of expected values for the characteristics of the respondents, such as gender, age, country of origin, level of education, marital status, and number of children, which the respondents have in relation to their confidence in vaccines. The analysis of the variance of the respondents’ data on the above characteristics, given in the previous table, has shown that the expected value for each individual characteristic (except for the state) differs in relation to the attitudes of parents, and that the given characteristics have a significant influence on the formation of attitudes. Based on the obtained results, it is possible to accept hypothesis H1.

Using the conclusion of hypothesis H1, and before testing the justification of the claim of hypothesis H2, it was tried to answer the question of the relationship between the demographic characteristics of respondents (who were the subject of hypothesis H1) and their negative attitude towards vaccines based on information from online media (correlated with hypothesis H2) and for that purpose logistic regression was applied. So, in the continuation of the research, we analyzed the influence of certain characteristics of parents on their attitude not to vaccinate a child, built on the content, which they found by consulting online media. The aim of this part of the analysis was to determine whether there is a certain group of respondents who are more vulnerable to content on online media and, on that basis, refuse to vaccinate a child. In order to determine the relationship between a particular characteristic of respondents and their attitude not to vaccinate a child, under the influence of information found on online media, as mentioned above, we used logistic regression because it is most often used to rank the relative importance of independent variables and to quantify the effect their interactions.

In order to define the variable which represents the negative attitude of parents towards vaccines, formed on the basis of content from online media, we chose the answer to one key question in the survey, which represents this behavior. To define the anti-vaccine attitude, we considered the answer to the question “Texts on online media about the negative effects of the vaccine affect the formation of my attitude to a significant extent.” because we believe that other questions about the negative attitude towards vaccines formed on the basis of online media are less focused on forming an attitude and making the final decision not to vaccinate the child. The independent variables in the model are the following key characteristics of the respondents: gender, age, country of origin, level of education, marital status, and number of children in the family. Thus, by assessing logistic regressions, we tried to find an answer to the question of whether men or women, younger or older parents, parents with higher or lower education, etc., are more prone to negative attitudes towards vaccines based on information obtained from online media. The results of the analysis are given below.

Before analyzing the model, we examined its quality by testing the hypothesis that there is no relationship between the dependent and independent variables in logistic regression. The test results are given in the following table (Table 4).

In this case, we tested the model by comparing the initial value of the logarithm, i.e., the model without an independent variable, which is 2896.054 with the final model, i.e., the model with an independent variable, which is 2401.083. With 72 degrees of freedom, χ2 is 494,971, which is significant at the level of 0%. The obtained results show that the model is meaningful and that the null hypothesis about the non-existence of a connection between the independent and dependent variables cannot be accepted.

The results of the evaluation of the logistic regression model are given in the following table (Table 5).

At the very beginning, it should be emphasized that all parameters in the regression are statistically significant with a risk of error of 5%. Since we are most interested in commenting on the results of extreme values on the Likert scale, we defined the value 1 as a basis for comparison (I completely disagree), and the value to explain the relationship between demographic characteristics and anti-vaccine attitude based on information from online media was defined with value 5 on the Likert scale (I completely agree). Based on the results of the estimated logistic regression, it is concluded that, if the parent-respondent is a male, i.e., the father, in 69.7% of cases, he will less often form a negative attitude towards vaccines based on information or texts read on online media compared to mothers-respondents. In other words, negative texts about vaccines through online media are not key to forming a negative attitude among fathers-respondents. If the age of the respondents’ parents is observed, conclusions on this issue are made on the basis of the reference group of parents, who are older than 45 years. If parents are under the age of 35, they are more likely to form a negative attitude towards vaccines and eventually make the decision not to vaccinate their child by reading texts through online media compared to parents over the age of 45. On the other hand, parents who are between 35 and 45 years old are about 80% less likely to form a negative attitude towards vaccines based on information from online media compared to the parents of the oldest age group of respondents in this survey. Parents from Serbia and Montenegro have between 15 and 18% less chance of forming a negative attitude towards vaccines reading articles on online media compared to parents from Bosnia and Herzegovina. Parents who have any lower level of education than doctors of science, are more likely to form a negative attitude towards vaccines based on information from the online media compared to parents with the title of doctor of science. This conclusion makes sense because parents with the highest level of education are more inclined to check the information and thoroughly process each topic before making such an important decision, such as vaccinating children. Parents who are married, in common law marriage, or divorced are more likely to build a negative attitude towards vaccines by reading content on online media compared to parents who are widowed. And that chance is incomparably higher for parents who are in common law marriage compared to other marital statuses. Finally, if the number of children in a family is observed, parents with less than three children are between 50% and 80% less likely to form a negative attitude towards vaccines based on texts they found on the Internet than parents with three or more children. This conclusion is connected with the fact that parents with a smaller number of children will strive to obtain additional information about vaccines from other sources, so the position on vaccines will not be formed only on the basis of content from the Internet, while parents with more children have a different situation.

Based on this part of the research it may be concluded that the following groups of parents are particularly vulnerable to the influence of online media on attitudes toward vaccines: women, parents of younger age (“millennials”), and parents who are in common law marriage, as well as parents who have more children.

Furthermore, in order to determine the level of influence that online media have on parents’ attitudes, an ANOVA test was applied to test the second hypothesis. The results are given in Table 6.

An analysis of the equality of expectancies for the characteristics of research on parents’ attitudes toward vaccines in the situation when using online media as a source of information has shown that respondents’ attitudes differ significantly depending on which aspect is used as a source of information. The conclusion is that online media has a significant influence on the formation of parents’ attitudes toward the vaccination of children, which leads to the acceptance of the hypothesis H2. In addition, the logistic regression itself showed that there is a significant difference in the formation of a negative attitude towards vaccines in different demographic groups of respondents, which is formed on the basis of information from online media.

In order to further examine the importance of a particular way of using online media on formation of attitudes, an eta coefficient was used, in which the squared value represents a relative measure of association. The eta coefficient takes a value between 0 and 1 and represents the proportion of variance in the dependent variable explained by the independent variable. The formula for calculating the eta coefficient is:

$$\eta^{2}=\frac{\mathrm{SSeffect}}{\mathrm{SStotal}}$$

where:

SSeffect = the sum of squares for a given independent variable (factor);

SStotal = the total sum of squares for all factors, interactions, and errors in the ANOVA analysis.

If η^2^ is 0.01, then it indicates a small impact, while a moderate impact is indicated for a value of 0.06 and a large influence for a value of 0.14 or greater. The results of the eta coefficient are given in Table 7.

The analysis of the η^2^ coefficient has shown that the greatest value in the opinion of parents that the texts on the online media about the negative effects of the vaccine influence the formation of the parents’ attitude (η^2^ = 0.216). Then, there is the opinion that these texts are generally correct (η^2^ = 0.181), and thirdly, is the view of parents that these texts have no scientific basis (η^2^ = 0.167), that is, they are based on fears and speculations. On the other hand, when claiming that the texts on the online media about negative effects have more importance than they should have, the value of the η^2^ coefficient is significantly 0.090.

In order to examine whether there are differences in the analyzed countries regarding the influence of online media on the formation of attitudes, additional testing has been done and the results are shown in Table 8.

The analysis of variance showed that the attitudes of the interviewed parents were the same, regardless of whether the respondents were from Montenegro, Serbia, or Bosnia and Herzegovina. Namely, the error made by rejecting the hypothesis that parents’ attitude toward trust in vaccines is equal to 98.7%, which is why we cannot reject it. In addition, in the section that follows (Table 9), an analysis of the eta squares for states in relation to attitudes, i.e., trust in vaccines, is presented.

The measure of association, that is, the eta coefficient for the country from which respondents come in relation to respondents’ attitudes toward vaccination is 0. In other words, there is no statistically significant difference between the analyzed countries for explaining the influence of online media on parents’ attitudes toward vaccines. Based on the results obtained previously, it may be concluded that hypothesis H3 is confirmed. Hence, the ANOVA test and the eta coefficient confirmed that the country of origin of the respondents does not affect their attitude towards the vaccination of children.

## 4. Discussion

Studies show that immunization, one of the greatest public health achievements, is occasionally hampered by strong biological, social, and cultural reactions of the public [68], which is why the media and communication are extremely important for this issue. In such conditions, online media, which today have become the primary source of information, are of special importance, especially because of the fact that many parents receive information about vaccinations mostly through online sources [10]. However, despite the growing literature on vaccination and the role of online media in modern times, and thus in the field of medicine and public health, we are still trying to explore and understand parents’ attitudes about vaccinating children, as well as why and how parents have different levels of trust in vaccines. This is particularly important given the research by Larson et al. [44], who found that socioeconomic status, media information, and attitudes and motivations regarding health care, as well as knowledge and awareness of the need for vaccines, are related to indecision to give the vaccine. This is especially present in less developed countries.

Thence, research on the impact of online media on parents’ attitudes towards child vaccination is, according to the authors’ knowledge, the first integrated scientific study in three countries of the Western Balkan region—Montenegro, Serbia, and Bosnia and Herzegovina. Therefore, the aim of the research was to investigate the level of influence that online media, as a form of marketing communication, have on the formation of attitudes toward the vaccination of children, in order to direct effectively and efficiently the potentials of this form of communication towards the strengthening and improvement of public health. Discussion of achieved results is presented below.

The obtained results confirmed the hypothesis that the identified characteristics of the respondents (gender, country of origin, age, marital status, and number of children) have a significant influence on attitudes towards vaccination of children, which is correlated with the results of other studies [69] in which demographic characteristics stand out as predictors of vaccines. Furthermore, the contingency analysis showed that the majority of respondents have high confidence in vaccines, when their characteristics, such as gender, country of origin, age, marital status, and number of children would be analyzed. The next largest category are parents who have a neutral attitude, i.e., are indifferent to the vaccination of their children, because this category consists of 27.87% of respondents. In third place are parents who completely trust in vaccines, and they make up 18.9% of all surveyed parents. The penultimate category is that of parents with low confidence in vaccines, and they represent 15.32% of all surveyed parents. In the last place are parents who do not believe in the positive effect of vaccinating their children. They make up only 4.02% of the surveyed parents in this study. Based on the obtained results, we may conclude that more than half of the respondents have a high or complete level of confidence in vaccines. However, about one-fifth of respondents do not trust or have a low level of trust in vaccines, which is correlated with the results of other studies [22,23,24] which confirm that distrust leads to vaccination rejection. These results suggest that certain actions must be taken in order to improve the existing situation. In that sense, the implementation of social marketing may be of great importance in order to strengthen trust in vaccines. Thus, social marketing in the field of immunization has a general social character, that is, it implies the implementation of marketing strategies in order to achieve specific goals of behavior oriented to the common good. In other words, the adequate implementation of social marketing may influence the change of behavior, that is, it may influence the strengthening of trust in vaccines. Thus, social marketing becomes important factor of improving public health.

Furthermore, the analysis of the variance of the respondents’ data on the stated characteristics showed that the expected value for each individual characteristic (except for the country) differs in relation to the attitudes of the parents, i.e., the level of trust in vaccines. These results are in correlation with the results of other studies, in which it is pointed out that important factors of influence are gender, age, level of education, and knowledge about immunization [53,54,55,56].

Findings of the research have shown a strong link between online media and parents’ attitudes, which is correlated with the results of some studies in other countries [8,32]. Namely, the analysis of equality of expected values for the characteristics of the research on parents ‘attitudes towards vaccines in the situation when using online media as a source of information has shown that respondents’ attitudes differ significantly depending on which aspect of online media is used as a source of information. In accordance with the above, the results of the descriptive statistics have shown that 37.7% of cases used online health websites, then blogs and forums (33.6%), when collecting online data about vaccines and indicate that the two sources were the most trusted online media outlets. For 11.7% of respondents, Facebook is the primary online source for information about vaccines, while 4.2% prefer other social networks (Twitter, Instagram). The participation of parents who were more informed about the arguments about the pro-vaccine (49.3%) and arguments against the vaccination of children (50.7%) is almost equal, which makes sense, because all those who ask for additional information should want to know both positive and negative arguments on this topic. In collecting information on vaccination through online media, 53.2% of parents stated that their spouse was also involved, while 46.8% of respondents stated that they collected the information for themselves. When asked how they rate their understanding of the material they received through online media, 55.6% of respondents consider their understanding to be average and 16.8% have a complete understanding, while the remaining respondents are not satisfied with their understanding of the information they obtained online. The “pressure” of pro-vaccine campaigns was felt by 13.74% of parents, while 40.55% felt the “pressure” of anti-vaccine campaigns, and the remaining respondents did not feel the pressure of these campaigns. Lastly, it is interesting that only 11.48% of respondents share vaccine information with others online, while 88.52% do not, although they use online sources to inform themselves. These results may be explained by the fact that parents in the analyzed area still use “offline” sources of information to form attitudes about vaccines compared to online media. The most common source of information are doctors, i.e., pediatricians (27.4%), while online media are used in combination with information obtained from doctors or family members and friends. Since this is the way they get information, they still do not have the habit of sharing their views on vaccines to other parents in public, i.e., online. Another reason is that parents in this study stated that they felt “the pressure of the online campaign against vaccines” (39% of respondents), so they did not want to be part of that campaign and impose their views through online media on other parents.

Based on the results obtained through logistic regression, we may conclude that the following demographic groups of parents are especially vulnerable to the formation of attitudes about vaccines: women, because as mothers they are more sensitive and easier to “scare” them with certain texts about harmfulness of vaccines for the health of their children; parents of younger age (“millennials”) who are influenced by digital technologies and use them in all spheres of life, including raising children, and it is not surprising that they are the most vulnerable age group of parents, who under the influence of online media form a negative attitude towards vaccines; parents living in common law marriage, as well as parents with more children, one of the key reasons being that more children give little free time to parents to devote to researching and finding additional sources of information to form attitudes about vaccines, so they rely to online sources. Precisely these differences that exist in different demographic groups when forming a negative attitude towards vaccines show the following: parents regularly follow texts about the negative effects of vaccines through online media and, based on this information, form different attitudes towards vaccines (logistic regression, for example, showed that mothers are more likely to form a negative attitude towards fathers, etc.), then that parents look differently at texts from online media about the negative effects of vaccines and thus view differently the importance of this information in forming attitudes towards vaccines (“millennials” will be more often influenced by information from online media compared to parents of older generations, etc.), parents differently estimate the truth of texts from online media about the negative effect of vaccines on children’s health (parents with more children are more likely to think these texts are true compared to parents who have one or two children, etc.), and parents react differently to these texts and some of them believe that they are texts that are not based on scientific facts (parents with a doctorate), while there are parents who believe that the texts are quite true (parents with a lower level of education in in relation to doctors of science, etc.), but in the end there are differences in the impact of this information obtained from online media on the final formation of attitudes about vaccines for different demographic categories of parents.

On the other hand, the analysis of the η^2^ coefficient has shown that the greatest value in the opinion of parents that the texts on the online media about the negative effects of the vaccine influence the formation of the parents’ attitude (η^2^ = 0.216). Namely, the research confirmed that parents were mostly under pressure from the negative campaign about vaccines through the online media (39% of respondents). Then, the research showed that parents trust the information they receive from medical websites the most (51.4% of respondents). Finally, the dominant group in the study consisted of mothers (70.1%), i.e., females, and the results of the logistic regression indicated that mothers were more influenced by the online campaign against vaccines. The combination of these factors has led to the fact that online media really play a significant role in forming the negative attitudes of respondents towards vaccines. Thus, based on the applied methods, we may conclude that online media, as a form of marketing communication, has a significant influence on the formation of parents’ position on vaccination of children, that is, trust in vaccines.

On the other hand, the ANOVA test and the eta coefficient confirmed that the country of origin of the respondents does not affect their attitude towards the vaccination of children. However, based on logistical regression, we concluded that, despite the fact that parents who come from these three different countries (Serbia, Montenegro, and Bosnia and Herzegovina) have approximately the same attitudes towards vaccines, the influence of online media on their attitude is different. It turned out that parents from Bosnia and Herzegovina are somewhat more susceptible to forming a negative attitude towards vaccines under the influence of online media than parents from Serbia and Montenegro. One of the potential reasons for this result may be found in the demographic characteristics of the respondents by country. While the demographic structure of the surveyed parents in Serbia and Montenegro is approximately the same, in Bosnia and Herzegovina, certain deviations have been noticed, which primarily refer to the level of education. Namely, in Bosnia and Herzegovina, there is a smaller number of respondents with the highest education (1.7%) compared to respondents with other lower levels of education. The dominant education is high school (44.2%), which, according to the results of logistic regression, has the greatest chance of forming a negative attitude of parents towards vaccines based on online media.

Finally, the authors tried to investigate whether there was a difference in attitudes of parents coming from different countries, regarding the influence of online media on formation of attitudes. When it comes to potential differences in the analyzed countries, the results of the survey have shown that the attitudes of the parents are the same, regardless of whether the respondents are from Montenegro, Serbia, or Bosnia and Herzegovina (η^2^ = 0). This should not be surprising having in mind the fact that these are developing countries, which have relatively similar legislation in the field of vaccination, and that all of them seek to harmonize public health policies in accordance with European standards. Thus, based on the above analysis, it may be noticed that there is no high value of deviation per state in the relation of the analyzed variable.

Based on all the above, we may conclude that there are a number of factors that affect parents’ attitudes toward vaccinating children and that online media are an important factor that determines parental behavior. In addition, research has shown that social marketing may be an important determinant of strengthening trust in vaccines, as well as improving of public health.

## 5. Conclusions and Implications

Today, information technologies have changed the paradigm of communication between medical professionals and public. The wide availability of information through the penetration of the mass media has played a significant role in encouraging parents [70,71,72] to form attitudes based on facilitated access to the media, especially when it comes to online media.

Concerns about vaccination have become a global phenomenon and led to the search for answers to the question of who has the greatest influence on the parental attitude toward vaccination: medical professionals, internet, or family and social environment [73]. In this regard, the widespread availability of information through online sources plays a significant role in the formation of attitudes [74]. Accordingly, in recent years, the role of Facebook as a source of pro and antivaccine information has also been analyzed [75,76]. Hence, in this area, social marketing has become especially important, in which activities are aimed at changing or maintaining the behavior of people for the benefit of individuals and society as a whole.

A large number of studies have been published over the years on the degree of exposure to vaccines [18,19,20]. There is also research on the impact of certain forms of online media (e.g., different forms of social media) on parents’ attitudes towards child vaccination. However, although several studies have examined parents’ attitudes toward vaccination of children, few of them, according to the authors, have integrated research in the way given in this study. Thus, unlike most previous research on vaccines, in which arguments for or against vaccination were mainly emphasized, the authors wanted to determine if there was a correlation between online media and parents’ attitudes towards vaccination through analysis in three countries in the Western Balkans region (Montenegro, Serbia, and Bosnia and Herzegovina), bringing the topic in the context of the application of social marketing in order to strengthen trust in vaccines and improve public health.

The authors developed and empirically tested a model that examined the relationship between influencing factors and parents’ attitudes toward vaccination of children. The authors used advanced descriptive statistics, as well as the ANOVA method, which allows to determine the individual influences of the analyzed factors related to the attitudes of parents about the vaccination of children, especially through the prism of online media. Logistic regression was applied to obtain a more precise answer to the question of the relationship between the demographic characteristics of the respondents and their negative attitude towards vaccines based on information from the online media. Furthermore, in order to further examine the importance of a certain way of using online media on the formation of attitudes, i.e., parents’ trust in vaccines, the eta-coefficient was used. The analysis of the η^2^ coefficient showed that the greatest influence on the formation of attitudes, i.e., parents’ trust in vaccines, when we look at the online media, have texts about the negative effects of vaccines that may be found on such sources.

The study has shown that the analyzed variables from the model have a significant influence on parents’ attitudes toward vaccination of children, and that they strongly reflect on the level of confidence in vaccines. Further, the study has shown that their impact varied depending on the factor being observed.

These conclusions may create more implications for decision-makers.

Based on the achieved results, the authors suggest that decision-makers should pay more attention to contemporary forms of social marketing, as well as online media, in order to focus their potential more on improvement of public health, as well as to avoid the harmful impact that these forms of communication may have an opinion on vaccines. In a broader sense, the authors conclude that these forms of communication affect not only attitudes about vaccination but the improvement of public health, which opens space for further research on this topic.

Professional support must be present in all forms of application of online media and social marketing in the field of immunization as important segment of public health. In this regard, the provision of information by health professionals and the quality of their information are essential for the decision to vaccinate or otherwise [77,78], which is extremely important for communication through social media, which are very common today.

Decision makers have to be aware that positive attitudes are key to a high level of confidence and that it is necessary to integrate a number of factors in order to maximize the application of social marketing in the field of medicine and public health.

In addition to the practical, the authors believe that this paper has a significant theoretical contribution. Namely, these results, except expanding the base of empirical research on the application of online media and social marketing in order to strengthen the trust in vaccines, offer added value to the existing literature by analyzing this concept in different countries and according to different factors. Additionally, the analysis is brought into the context of improving public health, which makes the work a special value. Finally, this analysis goes beyond the national framework and presents an analysis in a multi-country context, thus contributing to theorizing on the topic of social marketing and online media in the field of medicine and public health in an international context.

Finally, having in mind actual debates on the far-reaching consequences that the world will suffer globally due to external shocks caused by the new Covid-19 corona pandemic, we believe that, in the future, more detailed analyses of the impact of online media on vaccine confidence may be conducted exactly in this area. Not only in less developed countries, but also in developing countries, it is evident that the effects of the pandemic of the new coronavirus Covid-19 will be manifested through direct influences on people’s perception and attitudes about vaccination, especially when it comes to vaccination of children. In order to avoid the negative effects that online media may produce, decision-makers need to develop adequate online communication and social marketing strategies, for which the findings offered in this study may be very helpful and useful.

## Figures and Tables

**Table 1:** Characteristics of respondents.

Gender	N	Weighted%	Country	N	Weighted %
Female	1117	70.1	Montenegro	705	44.3
Male	476	29.9	Serbia	520	32.6
			Bosnia and Herzegovina	368	23.1
**Age**			**Level of education**		
18–24	37	2.3	Primary school	0	0
25–29	163	10.2	Secondary school	610	38.3
30–34	388	24.4	College	174	10.9
35–40	648	40.7	Faculty	638	40.1
41–45	212	13.3	Specialist	102	6.4
More than 45	145	9.1	Master	52	3.3
			PhD	17	1.0
**Marital status**			**Number of children**		
Married	1429	89.7	1	588	36.9
Extracurricular union	87	5.5	2	719	45.1
Divorced	63	4.0	3	275	17.3
Widower	14	0.9	More than 3	0	0

**Table 2:** Table of contingency for attitudes, i.e., trust in vaccines in relation to the characteristics of the respondents.

Rate Your Level of Trust in Vaccines		Gender	Age	Country	Level of Education	Marital Status	Number of Children
I don’t trust	Mean	1.08	3.84	1.77	2.81	1.16	1.77
	N	64	64	64	64	64	64
	Std. Deviation	0.270	1.359	0.792	1.006	0.541	0.611
Low level	Mean	1.25	3.87	1.80	3.22	1.26	1.98
	N	244	244	244	244	244	244
	Std. Deviation	0.432	1.068	0.821	1.168	0.777	0.751
Neutral	Mean	1.27	3.43	1.78	3.23	1.11	1.81
	N	444	444	444	444	444	433
	Std. Deviation	0.442	1.243	0.790	1.214	0.381	0.682
High level	Mean	1.36	4.00	1.80	3.60	1.20	1.80
	N	540	540	540	540	540	540
	Std. Deviation	0.481	0.971	0.803	1.271	0.526	0.731
I trust completely	Mean	1.32	3.91	1.78	3.49	1.09	1.66
	N	301	301	301	301	301	301
	Std. Deviation	0.468	1.191	0.762	1.473	0.364	0.678
Total	Mean	1.30	3.80	1.79	3.39	1.16	1.80
	N	1593	1593	1593	1593	1593	1582
	Std. Deviation	0.458	1.148	0.793	1.287	0.516	0.712

**Table 3:** ANOVA test of determination of differences based on characteristics of respondents.

Variables			Sum of Squares	DF	Mean Square	F	Sig.
Gender * Attitudes	Between Groups	(Combined)	6.673	4	1.668	8.099	0.000
	Within Groups		327.095	1588	0.206		
	Total		333.768	1592			
Age * Attitudes	Between Groups	(Combined)	87.568	4	21.892	17.279	0.000
	Within Groups		2011.939	1588	1.267		
	Total		2099.508	1592			
Conutry * Attitudes	Between Groups	(Combined)	0.215	4	0.054	0.085	0.987
	Within Groups		1001.492	1588	0.631		
	Total		1001.707	1592			
Level of education * Attitudes	Between Groups	(Combined)	66.847	4	16.712	10.322	0.000
	Within Groups		2570.951	1588	1.619		
	Total		2637.798	1592			
Marital status* Attitudes	Between Groups	(Combined)	5.694	4	1.423	5.401	0.000
	Within Groups		418.487	1588	0.264		
	Total		424.181	1592			
Number of children * Attitudes	Between Groups	(Combined)	14.048	4	3.512	7.037	0.000
	Within Groups		787.024	1577	0.499		
	Total		801.073	1581			

Symbol * represents the combination of two variables.

**Table 4:** Model fitting test for logistic regression of negative attitude of parents towards vaccines formed on the basis of information from online media.

Model Fitting Information				
Model	Model Fitting Criteria	Likelihood Ratio Tests		
	−2 Log Likelihood	Chi-Square	df	Sig.
Intercept Only	2896.054			
Final	2401.083	494.971	72	0.000

**Table 5:** Logistic regression of negative attitude of parents towards vaccines formed on the basis of information from online media.

“Texts on Online Media about the Negative Effects of the Vaccine Affect the Formation of My Attitude to a Significant Extent”		B	Std. Error	Wald	DF	Sig.	Exp(B)
I completely agree	Intercept	−17.677	1.467	145.197	1	0	
	[Gender = Male]	−1.195	0.295	16.409	1	0.05	0.303
	[Gender = Female]	0 ^a^			0		
	[Age = 18–24]	0.116	0		1		1.122
	[Age = 25–29]	0.078	0.0056	194.005	1	0	1.082
	[Age = 30–34]	0.194	0.0113	294.745	1	0	1.214
	[Age = 35–40]	−2.145	0.535	16.09	1	0.06	0.117
	[Age = 41–45]	−1.296	0.482	7.225	1	0.07	0.274
	[Age = Older than 45]	0 ^a^			0		
	[Country = Serbia]	−0.152	0.0134	128.670	1	0.008	0.859
	[Country = Montenegro]	−0.189	0.017	123.602	1	0.007	0.828
	[Country = Bosnia and Herzegovina]	0 ^a^			0		
	[Level of education = High School]	1.291	0.098	173.540	1	0	3.637
	[Level of Education = College]	0.043	0.005	73.960	1	0.01	1.043
	[Level of Education = Faculty]	0.843	0.096	77.110	1	0	2.323
	[Level of Education = Specialist]	0.892	0.129	47.813	1	0.01	2.44
	[Level of Education = Master]	0.013	1.559	0.000	1	0	1.013
	[Level of Education = PhD]	0 ^a^			0		
	[Marital status = Married]	0.17	0.019	80.055	1	0.02	1.185
	[Marital status = Common law marriage]	2.458	0.145	287.361	1	0	11.684
	[Marital status = Divorced]	0.19	0.017	124.913	1	0	1.209
	[Marital status = Widowed]	0 ^a^			0		
	[Number of Children = 1]	−1.607	0.39	16.947	1	0	0.2
	[Number of Children = 2]	−0.635	0.352	3.255	1	0.071	0.53
	[Number of Children = 3]	0 ^a^			0		

^a^—this variable is et to zero because it is redundant.

**Table 6:** ANOVA test of determination of online media on parent’s attitudes.

Variables			Sum of Squares	DF	Mean Square	F	Sig.
I regularly read texts on the online media about the negative effects of vaccines * Attitudes	Between Groups	(Combined)	51.420	4	12.855	8.610	0.000
	Within Groups		2370.804	1588	1.493		
	Total		2422.223	1592			
Texts on online media about negative effects have more importance than they really should have. * Attitudes	Between Groups	(Combined)	194.612	4	48.653	39.038	0.000
	Within Groups		1979.137	1588	1.246		
	Total		2173.749	1592			
The articles on the online media about the negative effects of the vaccine are mostly correct * Attitudes	Between Groups	(Combined)	304.422	4	76.106	87.903	0.000
	Within Groups		1374.882	1588	0.866		
	Total		1679.304	1592			
Texts on the online media about the negative effects of the vaccine are mostly based on fears and speculations, not on scientific facts * Attitudes	Between Groups	(Combined)	396.950	4	99.238	79.795	0.000
	Within Groups		1974.927	1588	1.244		
	Total		2371.877	1592			
Texts on the online media about the negative effects of the vaccine influence the formation of my attitude to a significant extent * Attitudes	Between Groups	(Combined)	564.536	4	141.134	109.449	0.000
	Within Groups		2047.712	1588	1.289		
	Total		2612.249	1592			

Symbol * represents the combination of two variables.

**Table 7:** Measures of association of online media with attitudes toward vaccines.

Variables	η	η^2^
I continuously read texts on the online media about the negative effects of vaccines	0.146	0.021
Texts on online media about negative effects of vaccines have more importance than they should have	0.299	0.090
The articles on the online media about the negative effects of the vaccines are mostly correct	0.426	0.181
Texts on the online media about the negative effects of the vaccines are mostly based on fears and speculations, not scientific facts	0.409	0.167
Texts on the online media about the negative effects of the vaccines influence the formation of my attitude to a significant extent	0.465	0.216

**Table 8:** ANOVA test examinations of differences by analyzed countries.

Variables			Sum of Squares	DF	Mean Square	F	Sig.
Country * Attitudes toward vaccines/Level of trust in vaccines	Between groups	(Combined)	0.215	4	0.054	0.085	0.987
	Within Groups		1001.492	1588	0.631		
	Total		1001.707	1592			

Symbol * represents the combination of two variables.

**Table 9:** Measures of association of countries with attitudes of respondents.

Variables	Eta	Eta Squared
Country * Attitudes toward vaccines	0.015	0.000

Symbol * represents the combination of two variables.

## Appendix A. Survey: The Impact of Online Media on Parents’ Attitudes toward Vaccination of Children

Respected,

This survey aims to determine the impact of online media on the attitudes of parents about the vaccination of children. The research is conducted for scientific purposes, with the aim of improving the situation in the subject area. The questionnaire contains 20 questions. The research is anonymous, and the results will be observed at the aggregate level.

Thank you for your participation!

1. Gender
  - Male
  - Female
2. Age
  - 18–24
  - 25–29
  - 30–34
  - 35–40
  - 41–45
  - More than 45
3. Country
  - Montenegro
  - Serbia
  - Bosnia and Herzegovina
4. Level of Education
  - Primary school
  - Secondary school
  - College
  - Faculty
  - Specialist
  - Master
  - PhD
5. Marital status
  - Married
  - Extramarital union
  - Divorced
  - Widow
6. Number of Children
  - 1
  - 2
  - 3
  - 4 and more
7. Did you do your own research about vaccines?
  - Yes
  - No
8. How did you inform yourself about vaccines?
  - Doctors
  - Family/relatives
  - Professional journals
  - Books
  - Flyers/brochures
  - Internet/social networks
  - Mass media (TV, newspapers, etc.)
  - Friends/associates
  - Other (please specify) _____________________
9. Which internet resources did you use when informing about vaccines?
  - Facebook
  - Other social media (Twitter, Instagram, etc.)
  - Wikipedia
  - Blogs/forums
  - Medical web sites
  - Other web resources (please specify) _________________
  - I didn’t use internet resources
10. Was your spouse included in collection of data about vaccination through online media?
  - Yes
  - No
11. Please rate your understanding of the materials that you collected through online media:
  - Little or no understanding at all
  - Poor understanding
  - Average understanding
  - Above average understanding
  - Complete understanding
12. During the research on online media, did you inform more about
  - Arguments about pro-vaccine
  - Arguments against vaccine
13. On the basis of information collected through online media, did you ask your child’s pediatrician to provide you information about vaccination and/or education?
  - Yes
  - No
14. In which format the pediatrician presented you information?
  - Conversation
  - Brochures/informative sheets
  - Suggested web pages/online sources
  - Referring to media
  - Other (please specify)
15. Do you share information about vaccines with other on social media?
  - Yes
  - No
16. Have you ever felt “the pressure” from pro- or anti-vaccine campaigns?
  - Yes, I have felt the “pressure” from pro-vaccines campaigns
  - Yes, I have felt the “pressure” from anti-vaccines campaigns
  - No, I haven’t felt the “pressure” from pro-vaccines campaigns
  - No, I haven’t felt the “pressure” from anti-vaccines campaigns
17. Rate your level of confidence in vaccines
  - I don’t believe at all
  - Low level of trust
  - Neutral
  - High level of trust
  - I really completely believe
18. Please rate your attitude about following statements about vaccination:
  - Vaccination is necessary in order to prevent illnesses
  - I believe my pediatrician
  - Vaccinations should be individual choice of parents
  - Immunity of illness is better than immunity of vaccination
  - Vaccination is ideal: once vaccinated, children may not get the illness against which they were vaccinated
  - It is necessary to have as more vaccinations as possible
  - Without vaccination, a child may become ill and, consequently, cause others to get it.
  - Vaccines are also given for illnesses that children probably will not get.
  - Vaccination is mostly safe for children
  - Vaccines consist of harmful substances
  - Plan of vaccination includes too many vaccines at the same time
  - Children get more vaccines than is useful for them
  - Vaccinations are harmful and they should be avoided
  - I am satisfied with the efforts that the State (relevant institutions) put forth regarding the vaccination

Note: Likert’s questions are rated on a scale from 1 to 5, where 1 = I completely disagree, 2 = I disagree, 3 = I cannot judge, 4 = I agree, and 5 = I completely agree.

19. Please rate your attitude about statements about the impact of online media about negative effects of vaccines
  - I regularly read texts in online media about negative effects of vaccines
  - Texts in online media about negative effects of vaccines significantly influence on my attitudes about vaccines
  - Texts in online media about negative effects of vaccines are mostly correct
  - Texts in online media about negative effects of vaccines are mostly based on fears and speculations, not on scientific facts
  - Texts in online media about negative effects of vaccines today have more importance than they deserve

Note: Likert’s questions are rated on a scale from 1 to 5, where 1 = I completely disagree, 2 = I disagree, 3 = I cannot judge, 4 = I agree, and 5 = I completely agree.

20. Which kind of online informing do you trust most:
  - Social networks
  - Medical web resources/sites
  - Blogs
  - Forums
  - Online newspapers and magazines
  - Other online resources ____________________

## References

[1] Camerini L., Diviani N., Tardini S. (2010). Health virtual communities: Is the self lost in the net?. Soc. Semiot..

[2] Percheski C., Hargittai E. (2011). Health information-seeking in the digital age. J. Am. Coll. Health.

[3] Vaterlaus J.M., Patten E.V., Roche C., Young J.A. (2015). #Gettinghealthy: The perceived influence of social media on young adult health behaviors. Comput. Hum. Behav..

[4] Fox S., Duggan M. Health Online Pew Internet & American Life Project, 2013, 1–4. http://www.pewinternet.org/~/media/Files/Reports/PIP_HealthOnline.pdf%5Cnhttp://www.pewinternet.org/2013/01/15/health-online-2013/#.

[5] Eysenbach G., Powell J., Kuss O., Sa E.R. (2002). Empirical studies assessing the quality of health information for consumers on the world wide web. JAMA.

[6] Ghenai A. (2017). Health misinformation in search and social media. Proceedings of the 2017 International Conference on Information Technology–ICIT December, 2017.

[7] Oyeyemi S.O., Gabarron E., Wynn R. (2014). Ebola, Twitter, and misinformation: A dangerous combination?. BMJ.

[8] Piscaglia L. (2016). Internet and Social Media: Influence on the Parent’s Vaccination Decision.

[9] Kao C.M., Schneyer R.J., Bocchini J.A. (2014). Child and adolescent immunizations. Curr. Opin. Pediatr..

[10] Heikkinen T., Tsolia M., Finn A. (2013). Vaccination of healthy children against seasonal influenza. Pediatr. Infect. Dis. J..

[11] Centers for Disease Control and Prevention (2010). Immunization Coverage in the U.S. Centers for Disease Control and Prevention. http://www.cdc.gov/vaccines/stats-surv/imz-coverage.htm.

[12] Weycker D., Edelsberg J., Halloran M.E., Longini I.M., Nizam A., Ciuryla V., Oster G. (2005). Population-wide benefits of routine vaccination of children against influenza. Vaccine.

[13] Jordan R., Connock M., Albon E., Frysmith A., Olowokure B., Hawker J., Burls A. (2006). Universal vaccination of children against influenza: Are there indirect benefits to the community? A systematic review of the evidence. Vaccine.

[14] Mirelman A.J., Ballard S.B., Saito M., Kosek M., Gilman R.H. (2015). Cost-effectiveness of norovirus vaccination in children in Peru. Vaccine.

[15] Shakerian S., Lakeh M.M., Esteghamati A., Zahraei S.M., Yaghoubi M. (2015). Cost-effectiveness of rotavirus vaccination for under-five children in Iran. Iran. J. Pediatr..

[16] Feikin D.R., Flannery B., Hamel M.J., Stack M., Hansen P.M., Black R.E., Walker N., Laxminarayan R., Temmerman M. (2015). Vaccines for children in low and middle-income countries. Reproductive, Maternal, New–Born, and Child Health: Disease Control Priorities.

[17] Larson H.J., De Figueiredo A., Xiahong Z., Schulz W.S., Verger P., Johnston I.G., Cook A.R., Jones N.S. (2016). The state of vaccine confidence 2016: Global insights through a 67-country survey. EBioMedicine.

[18] Smith J.C., Appleton M., Macdonald N. (2013). Building confidence in vaccines. Hot Topics in Infection and Immunity in Children IX.

[19] Larson H., de Figueiredo A., Karafillakis E., Rawal M. (2018). State of Vaccine Confidence in the EU 2018.

[20] Nowak G., Cacciatore M.A. (2016). Parents’ confidence in recommended childhood vaccinations: Extending the assessment, expanding the context. Hum. Vaccines Immunother..

[21] Jung M., Lin L., Viswanath K. (2015). Effect of media use on mothers’ vaccination of their children in sub-Saharan Africa. Vaccine.

[22] Yaqub O., Castle-Clarke S., Sevdalis N., Chataway J. (2014). Attitudes to vaccination: A critical review. Soc. Sci. Med..

[23] Larson H.J., Jarrett C., Eckersberger E., Smith D.M., Paterson P. (2014). Understanding vaccine hesitancy around vaccines and vaccination from a global perspective: A systematic review of published literature, 2007–2012. Vaccine.

[24] Jarrett C., Wilson R., O’Leary M., Eckersberger E., Larson H.J., SAGE Working Group on Vaccine Hesitancy (2015). Strategies for addressing vaccine hesitancy—A systematic review. Vaccine.

[25] Wakefield A.J. (1999). MMR vaccination and autism. Lancet.

[26] Parliament of Montenegro (2018). Law on Protection of the Population from Infectious Diseases. http://zakoni.skupstina.me/zakoni/web/dokumenta/zakoni-i-drugi-akti/327/1613-10375-28-2-17-3-4.pdf.

[27] Punished 150 Parents Who Did Not Vaccinate Their Children. https://medicalcg.me/11-februar-kaznjeno-150-roditelja-koji-nisu-vakcinisali-djecu/.

[28] Punished 150 Parents Who Did Not Vaccinate Their Children. https://www.adriaticnews.eu/2020/02/11/kaznjeno-150-roditelja-bez-vakcine-8-000-djece/.

[29] The World Health Organization (WHO) Ten Threats to Global Health in 2019. https://www.who.int/news-room/feature-stories/ten-threats-to-global-health-in-2019.

[30] Guillaume L.R., Bath P.A. (2004). The impact of health scares on parents’ information needs and preferred information sources: A case study of the MMR vaccine scare. Health Informatics J..

[31] Smith N., Graham T. (2017). Mapping the anti-vaccination movement on Facebook. Inf. Commun. Soc..

[32] Betsch C., Brewer N.T., Brocard P., Davies P., Gaissmaier W., Haase N., Leask J., Renkewitz F., Renner B., Reyna V.F. (2012). Opportunities and challenges of Web 2.0 for vaccination decisions. Vaccine.

[33] French J., Blair-Stevens C., McVey D., Merritt R. (2010). Social Marketing and Public Health: Theory and Practice.

[34] Flaskerud J.H. (2013). The nanny state, free will, and public health. Issues Ment. Health Nurs..

[35] Xu Z., Guo H. (2017). Using text mining to compare online pro- and anti-vaccine headlines: Word usage, sentiments, and online popularity. Commun. Stud..

[36] Jones A.M., Omer S.B., Bednarczyk R.A., Halsey N.A., Moulton L.H., Salmon D.A. (2012). Parents’ source of vaccine information and impact on vaccine attitudes, beliefs, and nonmedical exemptions. Adv. Prev. Med..

[37] Salmon D.A., Moulton L.H., Omer S.B., Dehart M.P., Stokley S., Halsey N.A. (2005). Factors associated with refusal of childhood vaccines among parents of school-aged children. Arch. Pediatr. Adolesc. Med..

[38] Tafuri S., Gallone M., Cappelli M., Martinelli D., Prato R., Germinario C. (2014). Addressing the anti-vaccination movement and the role of HCWs. Vaccine.

[39] Wakefield M., Loken B., Hornik R.C. (2010). Use of mass media campaigns to change health behaviour. Lancet.

[40] Dubé E., Vivion M., MacDonald N.E. (2014). Vaccine hesitancy, vaccine refusal and the anti-vaccine movement: Influence, impact and implications. Expert Rev. Vaccines.

[41] Grant L., Hausman B., Cashion M., Lucchesi N., Patel K., Roberts J., Koerber A., Lawrence H. (2015). Vaccination persuasion online: A qualitative study of two provaccine and two vaccine-skeptical websites. J. Med. Internet Res..

[42] Kata A. (2010). A postmodern Pandora’s box: Anti-vaccination misinformation on the Internet. Vaccine.

[43] Cameron C.D., Payne B.K. (2011). Escaping affect: How motivated emotion regulation creates insensitivity to mass suffering. J. Pers. Soc. Psychol..

[44] Larson H.J., Cooper L.Z., Eskola J., Katz S.L., Ratzan S. (2011). Addressing the vaccine confidence gap. Lancet.

[45] Bean S.J. (2011). Emerging and continuing trends in vaccine opposition website content☆. Vaccine.

[46] Quattrociocchi W. (2017). Part 2-Social and Political Challenges: 2.1 Western Democracy in Crisis? World Economic Forum [Internet]. http://reports.weforum.org/global-risks-2017/part-2-social-and-political-challenges/2-1-western-democracy-in-crisis/.

[47] Brown J., Broderick A.J., Lee N. (2007). Word of mouth communication within online communities: Conceptualizing the online social network. J. Interact. Mark..

[48] Quattrociocchi W., Caldarelli G., Scala A. (2014). Opinion dynamics on interacting networks: Media competition and social influence. Sci. Rep..

[49] Diekema D.S. (2005). Responding to parental refusals of immunization of children. Pediatrics.

[50] Ruiz J.B., Bell R.A. (2014). Understanding vaccination resistance: Vaccine search term selection bias and the valence of retrieved information. Vaccine.

[51] Davies P., Chapman S., Leask J. (2002). Antivaccination activists on the world wide web. Arch. Dis. Child..

[52] Zimmerman R., Wolfe R.E., Fox D., Fox J.R., Nowalk M.P.A., Troy J., Sharp L.K., Nasir L., Leask J. (2005). Vaccine criticism on the world wide web. J. Med. Internet Res..

[53] Brown K.F., Fraser G., Ramsay M.M., Shanley R., Cowley N., Van Wijgerden J., Toff P., Falconer M., Hudson M., Green J. (2011). Attitudinal and demographic predictors of measles-mumps-rubella vaccine (MMR) uptake during the UK catch-up campaign 2008–09: Cross-sectional survey. PLoS ONE.

[54] Anderberg D., Chevalier A., Wadsworth J. (2011). Anatomy of a health scare: Education, income and the MMR controversy in the UK. J. Health Econ..

[55] Walsh S., Thomas D.R., Mason B.W., Evans M.R. (2014). The impact of the media on the decision of parents in South Wales to accept measles-mumps-rubella (MMR) immunization. Epidemiol. Infect..

[56] Ristić M., Šeguljev Z., Petrović V., Vuleković V., Dugandžija T. (2013). The influence of sociodemographic characteristics of parents on immunization coverage of children. Opšta Med..

[57] Hoek J., Jones S.C. (2011). Regulation, public health and social marketing: A behaviour change trinity. J. Soc. Mark..

[58] Giubilini A. (2019). The Ethics of Vaccination.

[59] Braczkowska B., Kowalska M., Baranski K., Gajda M., Kurowski T.E., Zejda J. (2018). Parental opinions and attitudes about children’s vaccination safety in Silesian Voivodeship, Poland. Int. J. Environ. Res. Public Health.

[60] Hair J.F., Black W.C., Babin B.J., Anderson R.E., Tatham R.L. (2006). Multivariate Data Analysis.

[61] Tabachnick B.G., Fidell L.S. (2007). Using Multivariate Statistics.

[62] Fisher R.A. (1992). Statistical methods for research workers. Springer Series in Statistics.

[63] Fisher R.A. (1925). Statistical Methods for Research Workers.

[64] Rinker T. (2016). On the Treatment of Likert Data.

[65] Carifio J., Perla R. (2008). Resolving the 50-year debate around using and misusing Likert scales. Med. Educ..

[66] Norman G. (2010). Likert scales, levels of measurement and the “laws” of statistics. Adv. Health Sci. Educ..

[67] Hosmer D.W., Lemeshow S. (2000). Applied Logistic Regression.

[68] Wade G.H. (2014). Nurses as primary advocates for immunization adherence. MCN, Am. J. Matern. Nurs..

[69] Lee S., Riley-Behringer M., Rose J.C., Meropol S.B., Lazebnik R. (2016). Parental vaccine acceptance: A logistic regression model using previsit decisions. Clin. Pediatr..

[70] Tones K., Green J. (2004). Health Promotion: Planning and Strategies.

[71] Kar S.B., Pascual C.A., Chickering K.L. (1999). Empowerment of women for health promotion: A meta-analysis. Soc. Sci. Med..

[72] Ehrhardt A.A., Sawires S., McGovern T., Peacock D., Weston M. (2009). Gender, empowerment, and health: What is it? how does it work?. J. Acquir. Immune Defic. Syndr..

[73] Charron J., Gautier A., Jestin C. (2020). Influence of information sources on vaccine hesitancy and practices. Med. Mal. Infect..

[74] Lane S., Macdonald N.E., Marti M., Dumolard L. (2018). Vaccine hesitancy around the globe: Analysis of three years of WHO/UNICEF joint reporting form data-2015-2017. Vaccine.

[75] Gandhi C.K., Patel J., Zhan X. (2020). Trend of influenza vaccine Facebook posts in last 4 years: A content analysis. Am. J. Infect. Control..

[76] Owłasiuk A., Bielska D., Gryko A., Marcinowicz L., Czajkowski M., Kleosin K.N.P.H.C.I. (2018). Child vaccination programme in family doctor practices in 1997–2015: A cross-sectional study in Białystok, Poland. Pediatr. Med. Rodz..

[77] Czajka H., Czajka S., Biłas P., Pałka P., Jędrusik S., Czapkiewicz A. (2020). Who or what influences the individuals’ decision-making process regarding vaccinations?. Int. J. Environ. Res. Public Health.

[78] Lewandowska A., Lewandowski T., Rudzki G., Rudzki S., Laskowska B. (2020). Opinions and knowledge of parents regarding preventive vaccinations of children and causes of reluctance toward preventive vaccinations. Int. J. Environ. Res. Public Health.

